# Beyond HLA: an exploratory pilot study of non-HLA antibodies, HLA sensitization, and GSTT1 genotype in platelet transfusion refractoriness

**DOI:** 10.3389/fimmu.2026.1886771

**Published:** 2026-07-30

**Authors:** Jun Qi, Huachao Zhu, Manni Wang, Tianju Wang, Yuhui Li, Lixia Shang, Le Chen, Xiaofang Wang, Jing Shao, Chaofeng Ma, Hua Xu

**Affiliations:** 1 HLA Laboratory, Shaanxi Province Blood Center, Institute of Xi’an Blood Bank, Xi’an, Shaanxi, China; 2 Department of Hematology, The First Affiliated Hospital of Xi’an Jiao Tong University, Xi’an, Shaanxi, China

**Keywords:** colony stimulating factor 2, glutathione S-transferase theta-1, HLA alloimmunization, multiple transfusions, non-HLA antibodies, platelet transfusion refractoriness

## Abstract

**Objectives:**

Platelet transfusion refractoriness (PTR) is a common and clinically challenging condition with complex underlying mechanisms. Human leukocyte antigen (HLA) alloimmunization is the principal cause of immune PTR (iPTR), whereas non-HLA antibodies (non-HLA Abs) are known to contribute to antibody-mediated rejection, microvascular inflammation, interstitial fibrosis, and graft loss. However, whether non-HLA Abs contribute to the development of PTR and how they are associated with HLA alloimmunization remain unclear.

**Methods:**

This retrospective study evaluated the expression profiles of 60 non-HLA Abs and the degree of HLA alloimmunization in 32 patients with PTR and 20 healthy blood donors using a multiplex Luminex single-antigen bead assay. *GSTT1* gene deletion status in this cohort was determined by quantitative PCR (qPCR). Risk factors associated with the development of non-HLA Abs in PTR patients were analyzed using multivariable logistic regression with penalized maximum likelihood estimation (Firth correction).

**Results:**

The overall positivity rate of non-HLA Abs in PTR patients was significantly higher than that in healthy blood donors (90.63% vs. 55%, *p* = 0.006). Among the 14 positive non-HLA Abs tested, none showed statistically significant differences in odds ratios (ORs) between the non-iPTR and iPTR groups. Compared with healthy donors, only the positivity rates of CSF2 Ab and GSTT1 Ab were significantly increased in the non-iPTR and/or iPTR groups. A history of multiple transfusions was strongly associated with overall non-HLA Ab positivity, as well as with CSF2 Ab and GSTT1 Ab positivity (OR = 6.12, *p* = 0.008; OR = 21.9, *p* = 0.001; OR = 12.56, *p* = 0.017). Overall, 26 of 52 individuals (50%) across all groups exhibited homozygous deletion of the GSTT1 gene (GSTT1*0/0), among whom 34.62% (9/26) developed GSTT1 Ab. Among PTR patients with GSTT1*0/0, 56.25% (9/16) were positive for GSTT1 Ab. In contrast, no GSTT1 Ab were detected in healthy donors, although 45% (9/20) had a history of pregnancy.

**Conclusions:**

In this exploratory pilot study, non-HLA antibody profiles were independently correlated with PTR beyond HLA sensitization, with multiple transfusions as a clinically relevant factor linked to CSF2 and GSTT1 Abs, whereas the GSTT1*0/0 genotype appeared to be a prerequisite for the formation of GSTT1 Abs. Whether incorporating non-HLA antibody screening and GSTT1 genotyping into pre-transfusion evaluation can improve precision transfusion remains to be validated in prospective studies.

## 1 Introduction

Platelet transfusion refractoriness (PTR) is a common and challenging clinical problem in patients requiring repeated transfusions, significantly impacting therapeutic efficacy and prognosis (1, 2). The mechanisms underlying PTR are complex and generally involve immune factors (immune PTR, iPTR), non-immune factors (non-immune PTR, non-iPTR), and mixed causes. Non-immune factors include infection, high fever, hypersplenism, ongoing bleeding, disseminated intravascular coagulation, medications, and platelet quality; however, these factors are not always readily identifiable. In clinical practice, the absence of detectable platelet-related antibodies is often used as an exclusion criterion for diagnosing non-iPTR. Humoral immunity mediated by HLA class I antibodies is widely regarded as the principal pathological mechanism of iPTR (3). Nevertheless, some patients with PTR do not exhibit HLA or other platelet-related antibodies. Even after the exclusion of non-immune factors, these patients still demonstrate poor responses to platelet transfusions, suggesting that additional, incompletely understood mechanisms may contribute to the pathogenesis of PTR (4).

Recent studies have demonstrated that non-HLA antibodies (non-HLA Abs) targeting various endothelial or tissue-associated antigens possess significant immunological relevance in solid organ transplantation (5, 6). These antibodies are closely associated with antibody-mediated rejection, graft dysfunction, and poor long-term outcomes in kidney, liver, heart, and lung transplantation (7–9). Even in transplant recipients lacking HLA antibodies, non-HLA Abs may still mediate immune injury and increase the risk of graft rejection (10, 11). However, evidence regarding the potential synergistic interaction between HLA and non-HLA antibodies remains inconsistent.

Platelets are increasingly recognized as heterogeneous, dynamic, and multifunctional cells that serve not only central roles in thrombosis but also important functions in immune regulation (12). Repeated transfusion of blood components, including platelets, increases the risk of alloimmune sensitization in recipients. Given that certain non-HLA Abs can directly induce endothelial injury and complement activation, several important questions arise: Do non-HLA Abs contribute to the development of PTR? Is the production of non-HLA Abs associated with HLA sensitization in iPTR? In patients without iPTR, do non-HLA Abs participate in the pathogenesis of PTR? To date, these questions remain largely understudied in the existing literature.

This retrospective study analyzed non-HLA Abs expression profiles in patients with PTR using a multiplex Luminex single-antigen bead assay. The objectives were to determine the prevalence and distribution of non-HLA Abs in HLA-sensitized and non-sensitized PTR cohorts, and evaluate whether interactions between HLA and non-HLA antibodies contribute to the development of PTR. Notably, our exploratory findings provide preliminary observational evidence for non-HLA-mediated alloreactivity extending from the field of transplantation to transfusion, suggesting the potential existence of shared immune mechanisms and laying a foundation for future interdisciplinary research on immunomodulatory strategies.

## 2 Materials and methods

### 2.1 Sample collection

Samples were collected between June and December 2025 from patients diagnosed with PTR who underwent platelet compatibility testing before prophylactic or therapeutic platelet transfusion PTR was defined as a failure to achieve an increment of ≥5×10^9^/L in platelet count 12~14 h after two consecutive transfusions of fresh, ABO-identical platelets (13). For each participant, 3~5 mL of peripheral blood was collected in EDTA-anticoagulated and coagulated tubes, respectively. Based on the presence of pre-existing HLA-specific antibodies, 16 patients were assigned to the iPTR group. To minimize confounding factors, 16 patients with non-iPTR were matched to iPTR patients by sex, age, pregnancy history, transfusion history, and underlying diseases for comparative analysis. A history of multiple transfusions was defined as prior receipt of ≥3 allogeneic blood components (red blood cells, platelets, or plasma), consistent with commonly used operational definitions in studies of PTR and alloimmunization. In addition, 20 healthy voluntary platelet donors were randomly selected as controls. Non-HLA Abs results were interpreted according to manufacturer-specified cutoff values. Written informed consent was obtained from all participants. The study was conducted in accordance with Helsinki Declaration and approved by the Ethics Committee of the Shaanxi Blood Center (No [2026].10).

### 2.2 Platelet antibody screening

Platelet antibodies were detected using the PAK-Lx Assay kit (LIFECODES, USA). Briefly, 5 μL of pretreated patient serum was mixed with 20 μL of reaction beads and incubated at room temperature with shaking in the dark. The plate was then washed four times by vacuum aspiration, followed by incubation with phycoerythrin (PE)-conjugated anti-human IgG according to the manufacturer’s instructions. After completion of the incubation steps, bead fluorescence signals were acquired using a Luminex LABScan^®^ 3D high-throughput flow analyzer (Luminex, USA). The assay detected antibodies against GPIIb/IIIa, GPIb/IX, GPIa/IIa (HPA-1 to HPA-5), GPIV, and HLA class I antigens. Mean fluorescence intensity (MFI) values were analyzed using MATCH IT! Platelet Antibody software (Immucor, USA), and results were interpreted in accordance with the manufacturer’s instructions.

### 2.3 Identification of HLA class I specific antibodies

HLA class I-specific antibodies were detected using the Single Antigen Class I Detection Kit (LIFECODES, USA). Briefly, 5 μL of pretreated patient serum was mixed with 20 μL of reaction beads and incubated with shaking at room temperature for 30 min. The plate was then washed twice using vacuum aspiration, followed by incubation with 25 μL of PE-conjugated secondary antibody according to the manufacturer’s instructions. After completion of the incubation steps, bead fluorescence signals were acquired using a Luminex LABScan^®^ 3D high-throughput flow analyzer. The assay covered 30 antigens corresponding to the HLA-A locus and 58 antigens corresponding to the HLA-B locus. MFI values of HLA class I antibodies were obtained using MATCH IT! analysis software (version 1.3.0). Results were interpreted based on the manufacturer’s instructions in combination with the cutoff values established by our laboratory, with MFI≥1000 defined as positive.

### 2.4 Detection of the non-HLA antibody profiling

Non-HLA Abs were detected using the LIFECODES Non-HLA Antibody Assay Kit (Immucor, USA). This assay is based on a single-antigen bead platform and detects 60 non-HLA antibodies (7). The corresponding target antigens and their full gene names are listed in **Supplementary Table 1**. All procedures were performed in strict accordance with the manufacturer’s instructions and are briefly described as follows. Briefly, 40 μL of antigen-coated bead mixture and 10 μL of patient serum were added to pre-wetted filter plate wells and incubated on a rotating platform. After washing, the beads were incubated with 50 μL of phycoerythrin (PE)-conjugated anti-human IgG antibody diluted 1:10. Following a final wash, the beads were resuspended and analyzed using the Luminex LABScan^®^ 3D high-throughput flow analyzer. Antibody positivity was defined as an MFI value reaching at least twice the manufacturer’s preset standardized cutoff and simultaneously exceeding three times the mean MFI of normal controls.

### 2.5 Detection of homozygous Glutathione S-Transferase theta-1 gene deletion

A total of 200 μL of peripheral blood was collected for genomic DNA extraction using a blood genomic DNA extraction kit (Kaishuo Biotech, China), according to the manufacturer’s instructions. DNA extraction was performed using the HF16 Super automated nucleic acid extraction system (MagCore^©^, Taiwan, China). The extracted DNA was subsequently used for quantitative real-time polymerase chain reaction (qPCR) analysis of *GSTT1* gene copy status. qPCR was performed using *GSTT1*-specific primers and probe as follows: forward primer, 5′-CCTTACTGGTCCTCACATCTCCTT-3′; reverse primer, 5′-CCCACAGCACTCACATGCA-3′; probe, 5′FAM-CTGACCTCGTAGCCATCACGGAGCTG-3′BHQ1. Simultaneously, the HBB gene was used as an internal reference control for co-amplification, with the following primers and probe: forward primer, 5′-GCTGGCCCATCACTTTGG-3′; reverse primer, 5′-TTGTGGGCCAGGGCATTA-3′; probe, 5′VIC-AAGAATTCACCCCACCAGTGCAGGC-3′BHQ1. PCR amplification was carried out using the TAKARA Premix Ex Taq reagent system (RR390) on an ABI 7500 real-time PCR instrument. Genotypes were determined according to the amplification results of *GSTT1* relative to the internal control gene *HBB*. Samples showing no *GSTT1* amplification signal with normal *HBB* amplification were classified as homozygous *GSTT1* deletion (GSTT1*0/0, null genotype), whereas samples with normal amplification of both *GSTT1* and *HBB* were classified as *GSTT1*-present genotype.

### 2.6 Assessment of the degree of HLA alloimmunization sensitization

Based on the HLA antibodies detected in patients with PTR, donor panel reactive antibody (DPRA) was estimated to determine the proportion of donors whose HLA antigens would be incompatible with sensitized patients. HLA-A, -B, and -C gene frequency data from a local HLA-typed platelet donor registry (n = 3,590; data not shown) were used to represent the potential donor population. DPRA represents the percentage of potential donors whose HLA antigens are unacceptable to the patient and serves as a quantitative measure of the degree of HLA alloimmunization and the associated risk of transfusion incompatibility. A DPRA value of ≥80% is generally considered indicative of high sensitization (14).

### 2.7 Statistical analysis

Baseline characteristics were summarized using descriptive statistics. Continuous variables are presented as medians with interquartile ranges (IQRs), and group comparisons were performed using the Wilcoxon rank-sum test. Categorical variables are reported as counts and percentages, and comparisons between groups were conducted using the χ² test or Fisher’s exact test, as appropriate. Logistic regression models were applied to evaluate the associations between individual non-HLA Abs and iPTR status (iPTR vs. non-iPTR), with effect sizes expressed as odds ratios (ORs) and corresponding 95% confidence intervals (CIs). Differences in the number of positive non-HLA Abs among the iPTR, non-iPTR, PTR, and healthy donor groups were assessed using Fisher’s exact test and visualized using bar plots. Stacked bar charts were used to illustrate the distribution of the GSTT1*0/0 genotype across the four groups. Spearman correlation analysis was conducted using the psych package in R, and *p*-values were adjusted using the Benjamini-Hochberg method to control the false discovery rate (FDR). Hierarchical clustering of variables was performed using Euclidean distance and Ward’s D2 linkage method. The results were visualized as a heatmap generated with the pheatmap package, with correlation coefficients shown in the lower triangle and significance levels indicated in the upper triangle (*** *p*<0.001, ** *p*<0.01, * *p*<0.05). Risk factors associated with the development of non-HLA Abs were analyzed using logistic regression with Firth’s penalized likelihood correction. All statistical tests were two-sided, and a *p*-value<0.05 was considered statistically significant. All analyses were performed using R software (version 4.4.1).

## 3 Results

### 3.1 Baseline characteristics of PTR patients

A total of 32 patients with PTR were included in the study. Based on the presence of preformed HLA-specific antibodies, 16 patients were classified as HLA antibody-positive PTR (iPTR group), with a mean DPRA of 83.90 ± 16.21%; among them, 11 patients were highly sensitized, with a mean DPRA of 92.40 ± 6.37%. The remaining 16 patients were classified as HLA antibody-negative PTR (non-iPTR group). No statistically significant differences were observed between the non-iPTR and iPTR groups with respect to sex, age, blood type, pregnancy history, transfusion history, or the distribution of underlying diseases (*p*>0.05), indicating comparable baseline immunological risk between the two groups. Only one patient in the iPTR group tested positive for HPA antibodies (**Table 1**).

**Table 1:** Baseline characteristics of non-iPTR and iPTR patients.

Characteristic	Non-iPTR group (n = 16)	iPTR group (n = 16)	*p*-value^a^
Gender, n (%)			0.710
Female	10 (63%)	11 (69%)	
Male	6 (38%)	5 (31%)	
Age, Median (Q1, Q3)	59 (49, 64)	57 (53, 66)	0.880
Blood type, n (%)			1.000
A	6 (38%)	7 (44%)	
AB	2 (13%)	2 (13%)	
B	4 (25%)	4 (25%)	
O	4 (25%)	3 (19%)	
Pregnancy history, n (%)	9 (56%)	11 (69%)	0.465
Multiple transfusion history, n (%)	16 (100%)	16 (100%)	1.000
Transplantation history, n (%)	0 (0%)	0 (0%)	1.000
Disease type, n (%)			0.394
Hematologic malignancies	11 (69%)	14 (88%)	
	AML (4), ALL (2), CML (1), MM (2), SAA (1), NHL (1)	AML (9), ALL (1), MDS (2), MM (1), SAA (1)	
Solid tumor	5 (31%)	2 (13%)	
HLA alloimmunization (DPRA, %)	0	83.90 ± 16.21	<0.001
HPA antibody, n (%)			1.000
Negative	16 (100%)	15 (94%)	
Positive	0 (0%)	1 (6.3%)	

a Pearson’s Chi-squared test; Wilcoxon rank sum test; Fisher’s exact test; iPTR, immune platelet transfusion refractoriness; HPA, human platelet antigen; AML, acute myeloid leukemia; ALL, acute lymphoblastic leukemia; CML, chronic myeloid leukemia; MM, multiple myeloma; SAA, severe aplastic anemia; NHL, non-Hodgkin lymphoma.

### 3.2 Distribution characteristics of the non-HLA Abs profile in PTR patients

**Figure 1:**
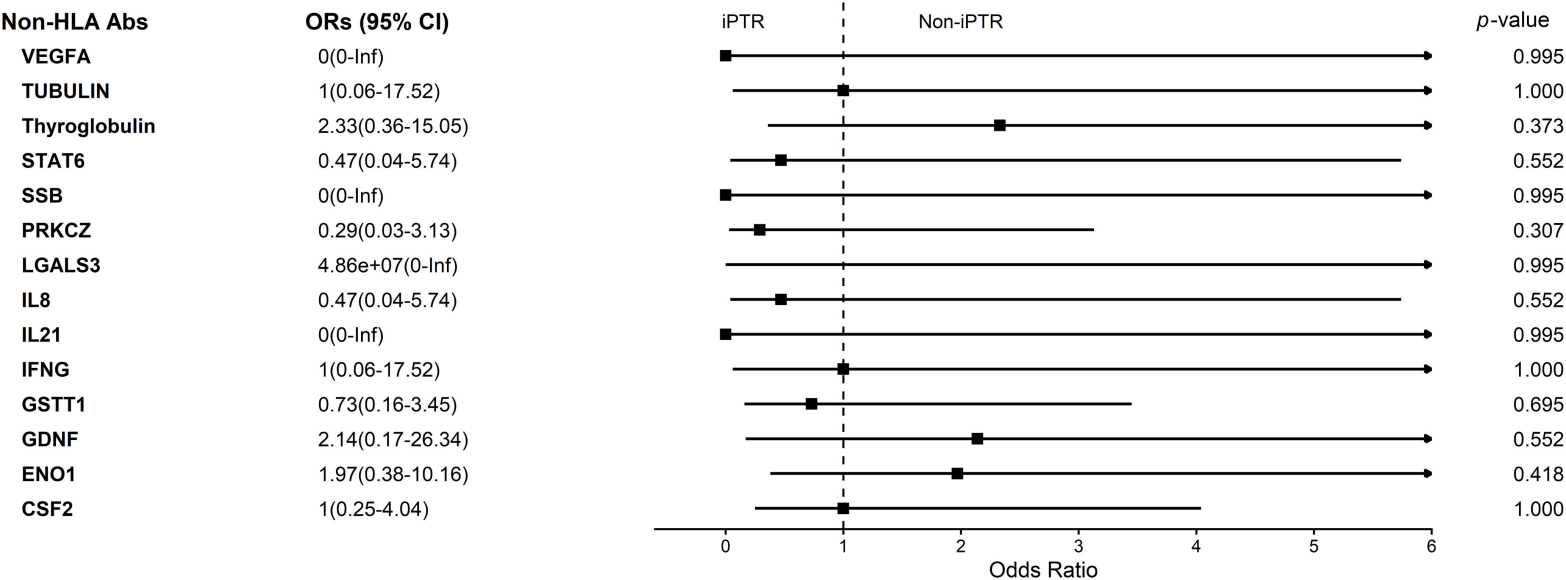
Forest plot of odds ratios for 14 non-HLA Abs in iPTR patients compared with non-iPTR patients. CI, confidence intervals; iPTR, immune platelet transfusion refractoriness; Non-HLA Abs, non-HLA antibodies; ORs, odds ratios.

A total of 60 non-HLA Abs were assessed using a multiplex Luminex single-antigen bead assay. Of these, 34 antibodies (CCP, CD40, COLLAGEN1, FIBRONECTIN1, KRT18, NCL, P2RY11, PTPRO, TUBA1B, ACTIN, AGRN, APOL2, ARHGDIB, COLLAGEN2, COLLAGEN3, COLLAGEN4, COLLAGEN5, COLLAGEN6, CXCL11, CXCL9, EMCN, GAPDH, HARS, HSPB1, KRT8, LGALS8, LMNA, MYOSIN, ROR1, SHC3, SNRPN, TUBB, VCL, and VIM) were not detected as positive in any PTR patients. An additional 12 antibodies (ATP5B, CGB5, DEXI, FAS, FLRT2, human transferrin, ICAM1, LPHN1, PECR, PLA2R1, PRKCH, and SNRPB2) were positive in only one case. Logistic regression analysis was subsequently performed on the remaining 14 non-HLA Abs to compare differences between non-iPTR and iPTR patients. The results indicated that none of the 14 antibodies showed statistically significant ORs, as illustrated in **Figure 1**.

### 3.3 Association between multiple transfusions and anti-colony stimulating factor 2 and anti-GSTT1 Abs

Baseline characteristics of healthy blood donors and total patients with PTR were summarized in **Table 2**. The median age was 43 years (Q1-Q3: 24~51) in healthy donors which were transfusion-naïve, significantly lower than 57 years (Q1-Q3: 53~65) in PTR patients (*p*<0.001). No significant differences between the two groups were observed in sex, blood type, or pregnancy history (*p*>0.05). It should be noted that, age did not differ significantly between patients who were positive or negative for anti-CSF2 Abs (median 56 vs. 52 years, *p* = 0.392) or between those who were positive or negative for anti-GSTT1 Abs (median 55 vs. 52 years, *p* = 0.271).

**Table 2:** Baseline characteristics of healthy blood donors and patients with PTR.

Characteristic	Controls	PTR	*p*-value^a^
	(n=20)	(n=32)	
Gender, n (%)			0.744
Female	14 (70%)	21 (66%)	
Male	6 (30%)	11 (34%)	
Age, Median (Q1, Q3)	43 (24, 51)	57 (53, 65)	<0.001
Blood type, n (%)			0.731
A	6 (30%)	13 (41%)	
AB	2 (10%)	4 (13%)	
B	8 (40%)	8 (25%)	
O	4 (20%)	7 (22%)	
Pregnancy history, n (%)	9 (45%)	20 (63%)	0.216
Multiple transfusion history, n (%)	0 (0%)	32 (100%)	<0.001

a Pearson’s Chi-squared test; Wilcoxon rank sum test; Fisher’s exact test; PTR, platelet transfusion refractoriness.

**Figure 2:**
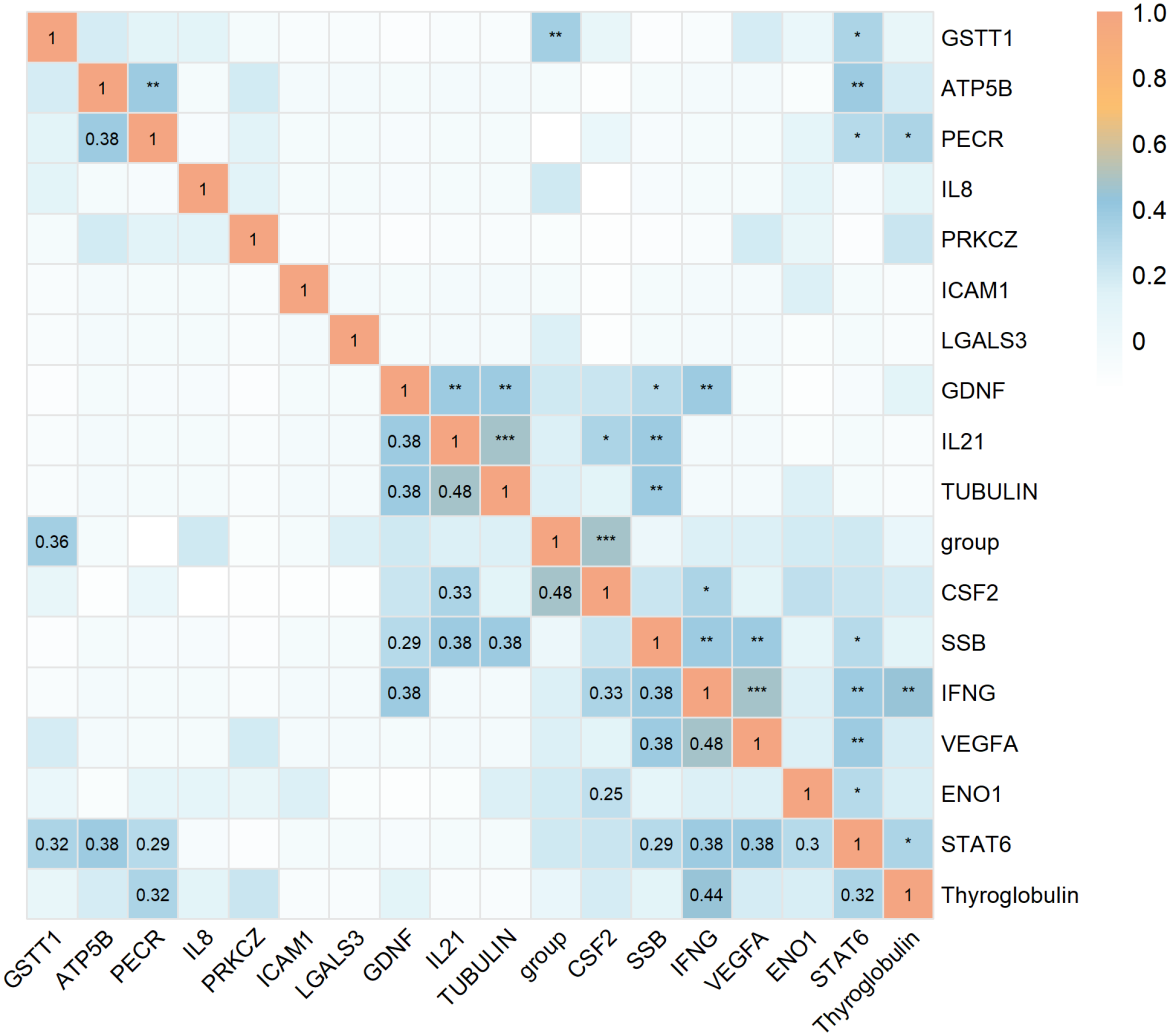
Correlation heatmap of positive non-HLA antibodies. The heatmap displays Spearman’s rank correlation coefficients (ρ). The lower triangle shows the correlation coefficients (values with |ρ| < 0.25 are omitted for clarity), whereas the upper triangle indicates significance levels based on Benjamini–Hochberg–adjusted p values (***p < 0.001, **p < 0.01, *p < 0.05). The color scale represents the strength and direction of the correlations: white indicates negative correlation, light blue indicates no correlation, and red indicates positive correlation. Groups: healthy blood donors and PTR patients.

The Spearman correlation analysis revealed predominantly weak to moderate positive correlations among non-HLA Abs (**Figure 2**). Among all antibody pairs, the strongest correlations were observed between IL21 and TUBULIN (ρ = 0.48) and between IFNG and VEGFA (ρ = 0.48); both remained statistically significant after correction for multiple comparisons (*p_c_* < 0.001). Hierarchical clustering identified several small antibody clusters with similar correlation patterns, including GSTT1–ATP5B–PECR; IL8–PRKCZ–ICAM1–LGALS3; IL21–TUBULIN–GDNF; and a larger cluster comprising CSF2–SSB–IFNG–VEGFA–ENO1–STAT6–Thyroglobulin (**Supplementary Figure 1**). The variable “group” (healthy blood donors *vs.* PTR patients) showed significant correlations only with CSF2 (ρ = 0.48, *p* < 0.001) and GSTT1 (ρ = 0.36, *p* < 0.01). Notably, no strong correlations (ρ > 0.50) were detected across the dataset, suggesting that although some antibodies show a degree of interrelatedness, most represent partially overlapping yet relatively independent features of the immune response.

**Figure 3:**
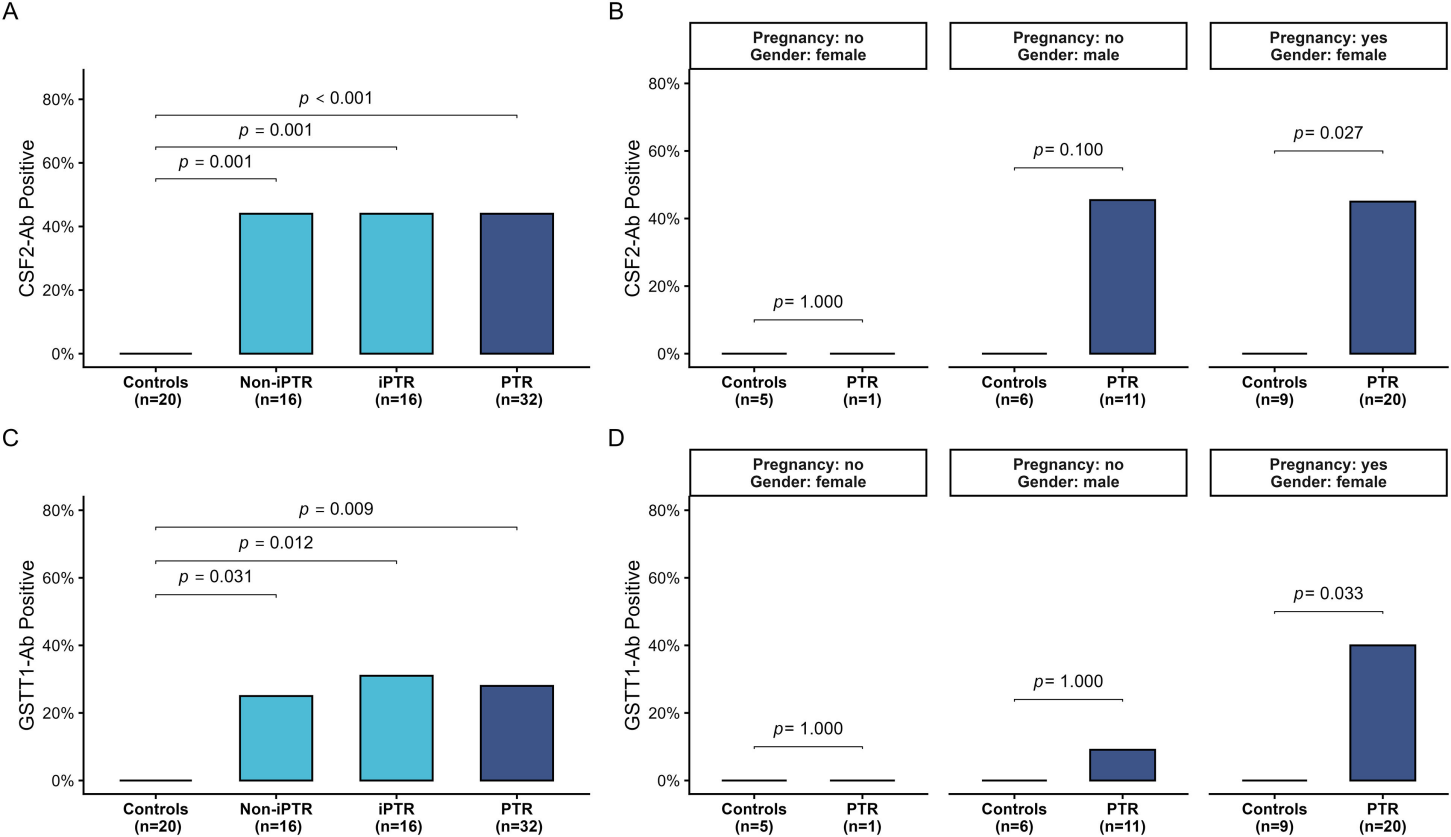
Distribution of Non-HLA Ab Positivity in PTR Patients and Healthy Blood Donors. **(A)** CSF2 Ab positivity rate. **(B)** CSF2 Ab positivity across subgroups stratified by pregnancy history and sex. **(C)** GSTT1 Ab positivity rate. **(D)** GSTT1 Ab positivity across subgroups stratified by pregnancy history and sex. CSF2 Ab, colony stimulating factor 2 antibody; GSTT1 Ab, Glutathione S-Transferase theta-1; iPTR, immune platelet transfusion refractoriness; PTR, platelet transfusion refractoriness.

The prevalence of each non-HLA antibody was compared between PTR patients and healthy blood donors, with the false discovery rate controlled using the Benjamini–Hochberg procedure (**Supplementary Table 2**). A significant difference between the two groups was observed in the positivity rate of CSF2 Abs (44% *vs*. 0%, *p* < 0.001, *p_c_* = 0.009). A difference was also observed for GSTT1 Abs (28% vs. 0%, *p* = 0.009, *p_c_* = 0.075), although this association did not remain significant after adjustment for multiple comparisons. No significant differences were identified for the other non-HLA antibodies. The positivity rate of CSF2 Abs was 43.75% (7/16, *p* = 0.0014) in both the non-iPTR and iPTR groups, and 43.75% (14/32, *p*<0.001) in the overall PTR patient group (**Figure 3A**). To identify risk factors for CSF2 Ab production, sex, pregnancy history, and history of multiple transfusions were included in a penalized maximum likelihood (Firth-corrected) logistic regression model. The analysis indicated that neither pregnancy history nor sex was significantly associated with CSF2 Ab positivity (OR = 2.24, 95% CI 0.1~347, *p* = 0.619; OR = 2.30, 95% CI 0.1~370, *p* = 0.618). In contrast, a history of multiple transfusions was strongly associated with CSF2 Ab positivity (OR = 21.9, 95% CI 2.76~2744, *p* = 0.001).

Similarly, the positivity rates of GSTT1 Abs were 25% (4/16, *p* = 0.031) in non-iPTR patients and 31.25% (5/16, *p* = 0.012) in iPTR patients, with an overall positivity rate of 28.13% (9/32, *p* = 0.009) among all PTR patients (**Figure 3C**). A penalized maximum likelihood (Firth-corrected) logistic regression model incorporating sex, pregnancy history, and transfusion history showed that neither pregnancy history nor sex was significantly associated with the presence of GSTT1 Abs (OR = 1.92, 95% CI 0.1~289, *p* = 0.682; OR = 0.41, 95% CI 0.01~71, *p* = 0.645). In contrast, a history of multiple transfusions was significantly associated with GSTT1 Ab positivity (OR = 12.56, 95% CI 1.45~1551, *p* = 0.017). Among women with a history of pregnancy, neither CSF2 nor GSTT1 Abs were detected in healthy blood donors without a transfusion history, whereas PTR patients with a history of multiple transfusions showed significantly higher positivity rates for both CSF2 and GSTT1 Abs (45%, *p* = 0.027; 40%, *p* = 0.033) (**Figures 3B, D**).

### 3.4 Association between multiple transfusions and overall non-HLA Ab positivity

**Figure 4:**
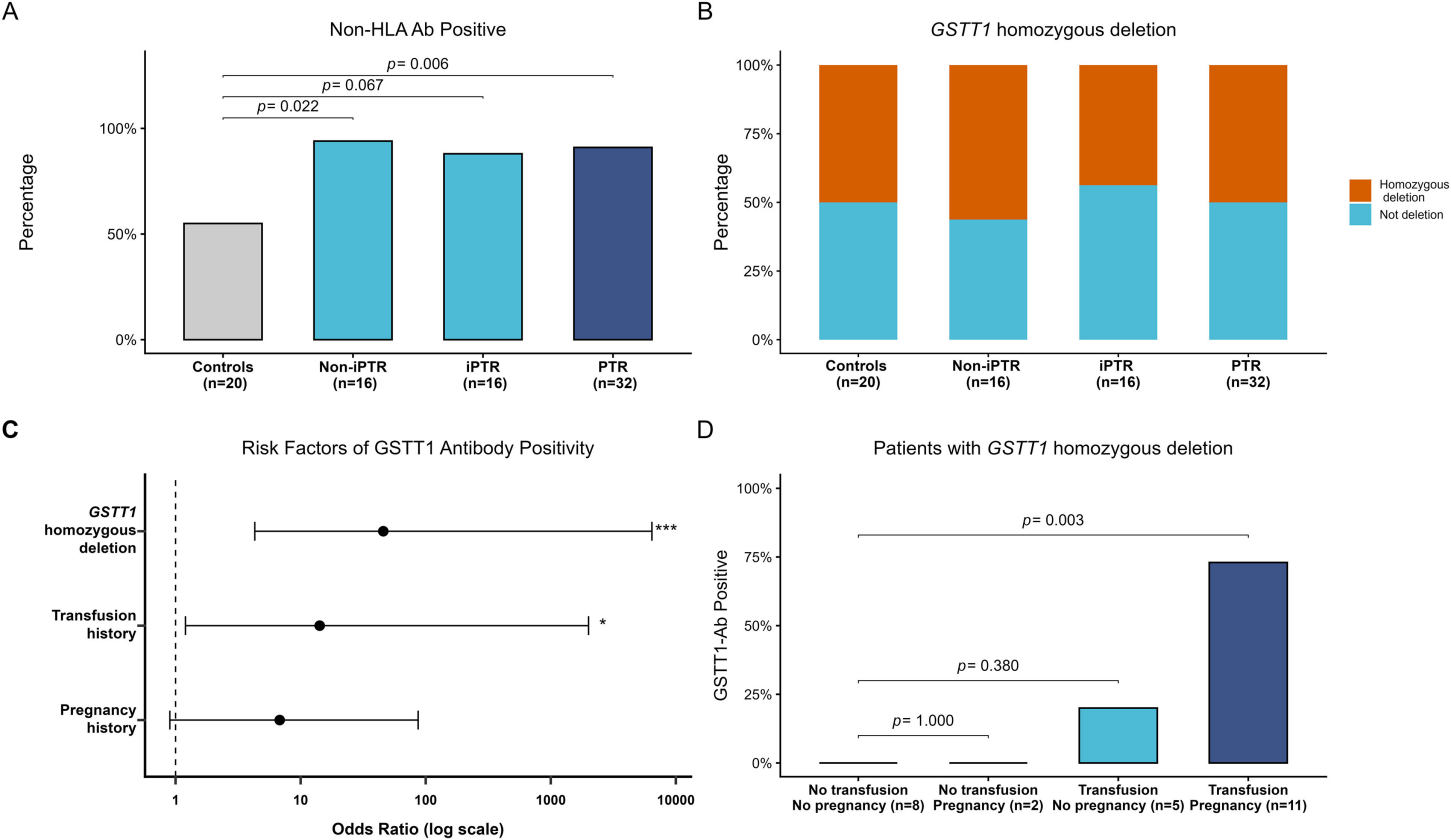
Overall positivity of non-HLA Abs and *GSTT1* homozygous deletion (GSTT1*0/0). **(A)** Overall prevalence of non-HLA Abs in PTR patients compared with healthy blood donors. **(B)** Stacked bar chart showing the proportion of individuals with GSTT1*0/0 among healthy blood donors, non-iPTR, iPTR, and PTR groups. **(C)** Factors associated with GSTT1 Ab positivity, presented as ORs with 95% CIs for GSTT1*0/0, transfusion history, and pregnancy history; asterisks indicate statistical significance (*p < 0.05; ***p < 0.001). **(D)** GSTT1 Ab positivity rates among subgroups of individuals with GSTT1*0/0, stratified by transfusion history and pregnancy history. GSTT1, Glutathione S-Transferase theta-1; iPTR, immune platelet transfusion refractoriness; PTR, platelet transfusion refractoriness.

Further analysis of overall non-HLA Ab positivity among patients with PTR classified individuals as positive if they tested positive for any non-HLA Ab; otherwise, they were classified as negative. The overall positivity rate of non-HLA Abs was 55% (11/20) among healthy blood donors and 90.63% (29/32) in the PTR group, indicating a statistically significant difference (*p* = 0.006). Compared with healthy blood donors, the positivity rates in the non-iPTR and iPTR groups were 93.75% (15/16, *p* = 0.022) and 87.50% (14/16, *p*>0.05), respectively (**Figure 4A**). In Firth-corrected logistic regression analysis, a history of multiple transfusions was significantly associated with overall non-HLA Ab positivity. Patients with a history of multiple transfusions had a markedly increased risk of testing positive for non-HLA Abs (OR = 6.12, 95% CI 1.59~28.5, *p* = 0.008). In contrast, a history of pregnancy was not significantly associated with the outcome (OR = 2.60, 95% CI 0.66~11.26, *p* = 0.173).

### 3.5 Multivariate logistic regression analysis of the association between *GSTT1* gene deletion and GSTT1 Ab formation

We further assessed the GSTT1 gene deletion status in all 52 individuals in the study cohort (**Supplementary Table 3**). Among them, 26 (50%) were identified as having the GSTT1*0/0 genotype. Both healthy blood donors and the overall PTR patient group showed a prevalence of 50% for GSTT1*0/0. The frequencies of GSTT1*0/0 were 56.25% (9/16) in the non-iPTR subgroup and 43.75% (7/16) in the iPTR subgroup, with no statistically significant differences observed among the groups (**Figure 4B**). In this study, GSTT1 Abs were detected exclusively in individuals with the GSTT1*0/0 genotype, with an overall positivity rate of 34.62% (9/26, *p* = 0.002). A penalized maximum likelihood (Firth-corrected) logistic regression model incorporating transfusion history, pregnancy history, and GSTT1*0/0 status showed that the GSTT1*0/0 genotype was significantly associated with an increased risk of GSTT1 Ab (OR = 45.8, 95% CI 4.3~6437, *p*<0.001). Similarly, compared with healthy blood donors without a transfusion history, PTR patients with multiple transfusions had a significantly higher risk of developing GSTT1 Ab (OR = 14.2, 95% CI 1.2~2004, *p* = 0.034). Pregnancy history (yes vs. no) showed a trend toward increased risk, although this association did not reach statistical significance (**Figure 4C**). Within the GSTT1*0/0 population, no GSTT1-Ab was detected in individuals with neither transfusion nor pregnancy history, nor in those with a pregnancy history but no transfusion history. In contrast, the positivity rate was 20% (*p* = 0.38) among individuals with a transfusion history but no pregnancy history, and 72.73% (*p* = 0.003) among those with both transfusion and pregnancy histories (**Figure 4D**).

## 4 Discussion

Non-HLA antibodies refer to alloantibodies or autoantibodies targeting tissue antigens other than HLA. Genetic disparities between donors and recipients can induce the production of non-HLA alloantibodies, whereas non-HLA autoantibodies may arise from the breakdown of immune self-tolerance or from tissue injury. Such injuries including ischemia-reperfusion injury, calcineurin inhibitor toxicity, alloimmune responses, and chronic inflammation—can promote the formation and exposure of cryptic antigens to the immune system (15). Increasing evidence indicates that non-HLA Abs are associated with antibody-mediated rejection (AMR) in kidney transplantation and may also influence graft survival (16). In addition to classical antigens such as HLA and HPA, platelets express a variety of transmembrane proteins and glycoproteins, some of which are also present on vascular endothelial cells and tissue epithelial cells. These molecules may therefore serve as potential targets of alloimmune responses (17).

To our knowledge, this is the first study to conduct a comparative, multiparametric profiling of non-HLA Abs across three clinically distinct populations—healthy donors, iPTR, and non-iPTR—offering an exploratory look into their differential expression patterns. The results showed that non-HLA humoral sensitization is common among PTR patients. Some non-HLA Abs exhibit a modest degree of correlation; however, most reflect partially overlapping yet largely independent immunological response profiles. Although differences in age and underlying disease backgrounds limit our analysis, we cannot definitively establish a causal relationship between these non-HLA Abs and PTR. However, based on our findings, we propose that the coexistence of these antibodies may reflect cumulative immune exposure or a state of immune dysregulation in PTR patients, including infections, inflammation, autoimmunity, and alloimmunity (18). Repeated transfusions, inflammatory cytokine release, and potential environmental or epigenetic influences may further predispose PTR patients to non-HLA Ab formation (19).

Several studies have suggested that non-HLA alloimmunization can occur independently of HLA alloimmunization and contribute to graft injury (20). Consistent with this notion, our study found no significant differences in non-HLA Ab profiles between the non-iPTR and iPTR groups (**Figures 1**, **3A, C**, **4A**), and no association between patterns of non-HLA Ab positivity and HLA DPRA. These findings support the concept that non-HLA Abs represent a distinct immunological axis (21, 22). However, the limited sample size and the low prevalence of certain antibodies may have reduced the statistical power to detect moderate effect sizes. Therefore, the lack of statistical significance does not preclude their potential biological relevance. Further analysis using a penalized maximum likelihood (Firth-corrected) logistic regression model identified a history of multiple transfusions as a strong independent risk factor for the development of anti-CSF2 and anti-GSTT1 Abs (OR = 21.9, *p* = 0.001; OR = 12.56, *p* = 0.017). These results suggest that persistent antigen exposure from repeated allogeneic transfusions may drive B-cell activation and alloimmune responses, thereby promoting the production of CSF2 and GSTT1 Abs. In contrast, gender and pregnancy history were not significantly associated with non-HLA Ab positivity, indicating that the sensitization pattern of non-HLA Abs differs from that of HLA antibodies and may depend more strongly on factors such as transfusion-related antigen burden and the local immune microenvironment. Overall, these findings provide a data-driven basis for risk stratification and the development of targeted desensitization or immunomodulatory strategies in PTR patients.

GSTT1 is a phase II detoxification enzyme involved in cellular defense and DNA damage repair. The *GSTT1* null genotype has been associated with altered capacity to scavenge free radicals, protect cells from oxidative stress, and detoxify harmful compounds. Deficiency of this enzyme may impair the recovery of DNA damage (23). GSTT1 is localized in the cytoplasm and is expressed in the liver, kidneys, pancreas, red blood cells, and other tissues, but not in lymphocytes (24). The *GSTT1* gene has two main variants: GSTT1*1/1 (wild-type) and GSTT1*0/0 (gene deletion), the latter resulting in absence of protein expression. Individuals with the GSTT1*0/0 null genotype may develop anti-GSTT1 Abs after exposure through pregnancy, blood transfusion, or transplantation (25, 26). The results of this study suggest that the GSTT1*0/0 genotype is a prerequisite for the production of anti-GSTT1 Abs. Among GSTT1*0/0 patients with PTR, GSTT1 Abs were detected in 56.25%. In contrast, no GSTT1 Abs were detected in GSTT1*0/0 healthy blood donors, although 45% had a history of pregnancy, suggesting that pregnancy alone may be insufficient to induce persistent GSTT1 Ab production.

Recent studies indicate that anti-GSTT1 Abs play a significant role in various alloimmune responses. Comoli et al. (15). and Solà-Porta et al. (27). reported that, among kidney transplant recipients without pre-existing HLA sensitization, elevated postoperative levels of anti-GSTT1 Abs were independently associated with AMR and graft loss. Similarly, the risk of acute transplant rejection was significantly higher in heart and lung transplant recipients who tested positive for anti-GSTT1 Abs (26). In hematopoietic stem cell transplantation (HSCT), GSTT1 is regarded as a minor histocompatibility antigen. Gene mismatch, particularly when the donor lacks GSTT1 and the recipient is GSTT1-positive, can induce specific humoral immune responses and is significantly associated with acute graft-versus-host disease (aGVHD) and graft failure (15). In *GSTT1*-null individuals, pre-sensitized GSTT1-specific memory B cells may arise following pregnancy or previous blood transfusions. Upon subsequent transfusion or transplantation from a GSTT1-positive donor, these memory B cells can be reactivated and produce antibodies. B cells not only recognize cytoplasmic GSTT1 released from damaged cells via the B-cell receptor (BCR) but may also sustain T-cell responses by functioning as antigen-presenting cells (APCs), providing cytokines and co-stimulatory signals required for the differentiation of long-lived memory T cells (28). However, in the present study, 43.75% of PTR patients with the GSTT1*0/0 genotype and a history of pregnancy or multiple transfusions did not develop detectable GSTT1 Abs or specific BCR. Notably, two of these patients were highly sensitized and exhibited broad, strongly positive HLA antibodies, suggesting that the development and underlying mechanisms of non-HLA Abs may differ from, or may not occur in synchrony with, those of HLA antibodies—a pattern also observed in organ transplantation (29).

In this study, significantly elevated levels of anti-CSF2 Abs were detected in PTR patients. The pro-inflammatory cytokine CSF2, also known as granulocyte–macrophage colony-stimulating factor (GM-CSF), plays an important role in immune cell differentiation, activation of the monocyte-macrophage system, and maintenance of tissue homeostasis. Anti-CSF2 Abs can neutralize CSF2 activity or interfere with receptor binding, thereby impairing its immunomodulatory and vasculoprotective functions and potentially indicating chronic inflammation and immune dysregulation (30). Previous studies have shown that elevated anti-CSF2 Ab levels are associated with atherosclerosis-related diseases and increased risks of vascular injury and cerebrovascular events (31). High pre-transplant levels of anti-CSF2 Abs in kidney transplant recipients may contribute to hyperacute rejection after transplantation (32, 33). As an inflammatory cytokine, CSF2 also plays a critical role in inflammatory and T-cell-mediated diseases, including the development of aGVHD (34). Our results also showed elevated anti-CSF2 Ab levels in patients with acute myeloid leukemia, chronic myeloid leukemia, and myelodysplastic syndromes, consistent with previous reports (35, 36). Overall, anti-CSF2 Abs may reflect systemic inflammatory status and disease progression, indicating their potential utility as biomarkers for immune risk assessment and inflammation monitoring.

On the other hand, non-HLA targets are predominantly immune-related, involving in endothelial and vascular immune stress, immune activation, co-stimulatory and pro-inflammatory signaling pathways (37). The pathogenic mechanisms of non-HLA Abs in organ transplantation may involve microvascular inflammation and endothelial injury (38). Anti-AT1R, anti-SNRPB, anti-GSTT1, and anti-Actin Abs can act synergistically with HLA antibodies and have been associated with T cell-mediated rejection (39,40), thereby further increasing immunological risk in transplantation. In this exploratory pilot study, whether the significantly elevated positive rate of non-HLA antibodies in iPTR and non-iPTR patients reflects enhanced platelet activation or excessive consumption *in vivo*, as well as the causal relationship between non-HLA antibodies and potential microvascular inflammation or tissue damage in PTR patients, remains to be elucidated through future mechanistic investigations.

This study has several limitations. First, we acknowledge the limited sample size as a limitation of this exploratory study. To address this issue, we applied Firth-corrected (penalized maximum likelihood) logistic regression. By introducing a penalty term into the likelihood function that shrinks regression coefficients, this approach reduces parameter bias and prevents model non-convergence associated with small sample sizes and rare outcomes. It also helps suppress overfitting and yields estimates of the associations between risk factors and non-HLA Ab production that are closer to the true values. Second, another major limitation of this study is the absence of functional validation, including platelet activation and aggregation assays, macrophage phagocytosis tests, and assessments of endothelial cell injury. The exploratory associations support the hypothesis that these factors may serve as biomarkers of immune dysregulation in PTR, rather than constituting definitive evidence of independent predictors. Moreover, its causal role in the pathogenesis of PTR has also not been established. Third, we acknowledge that multiple transfusions history is highly confounded with PTR status in the present cohort, as repeated transfusion is a major clinical predisposing factor for PTR. This inherent limitation should be considered when interpreting our correlational findings. In addition, unlike HLA antibody testing, non-HLA Ab detection lacks confirmatory strategies such as virtual crossmatching. Future research should include multicenter matched prospective studies with complete transfusion records and standardized data collection. Analytical approaches such as restricted cubic spline modeling should be applied to rigorously evaluate both linear and nonlinear dose–response relationships. In addition, cellular-level functional studies are needed to validate the clinical relevance of these associations and to elucidate the underlying immunological mechanisms.

This study lacked systematic evaluation of infection histories and viral loads for cytomegalovirus (CMV), Epstein–Barr virus (EBV), and varicella-zoster virus (VZV). Given that CMV and EBV seroprevalence exceeds 90% in Chinese adults (39), serology alone cannot distinguish the groups. CMV reactivation is a known risk factor for PTR and may confound both HLA and non-HLA antibody levels; evidence for EBV and VZV remains insufficient. Future studies require prospective viral DNA monitoring.

## 5 Conclusion

This exploratory study is the first to demonstrate, in a well-characterized Chinese cohort, that non-HLA antibody profiles are independently associated with PTR beyond HLA sensitization. A history of multiple transfusions was identified as a clinically relevant factor linked to the development of anti-CSF2 and anti-GSTT1 Abs, and the GSTT1*0/0 genotype was a prerequisite for the induction of anti-GSTT1 Abs. These findings suggest that such antibodies may serve as potential biomarkers of immune dysregulation. Their association with PTR could possibly reflect disturbances in B-cell tolerance and broad immune activation, rather than direct pathogenic effects. Establishing causality will require validation in independent large cohorts and additional *in vitro* functional studies.

Although HLA antibodies remain central to immunological risk assessment and immune monitoring in organ transplantation, accumulating evidence indicates that non-HLA antibodies also play important roles in AMR, microvascular inflammation, interstitial fibrosis, and graft loss. Whether incorporating non-HLA antibody screening and GSTT1 genotyping into pre-transfusion assessments can improve transfusion precision remains to be validated in prospective studies.

## Data Availability

The original contributions presented in the study are included in the article/**Supplementary Material**. Further inquiries can be directed to the corresponding author.
