# Supplementary material for: Beyond HLA: an exploratory pilot study of non-HLA antibodies, HLA sensitization, and GSTT1 genotype in platelet transfusion refractoriness
Source: Front Immunol. 2026 Jul 30;17:1886771. doi: 10.3389/fimmu.2026.1886771 (PMC13469656; doi:10.3389/fimmu.2026.1886771)
Supplement: Supplementary file 1 [file DataSheet1.docx]

**Supplementary Figure S1.** Hierarchical clustering dendrogram of non-HLA antibodies. Hierarchical clustering analysis was performed based on Spearman correlation patterns using Euclidean distance as the distance metric and Ward's D2 as the linkage method. The dendrogram illustrates the hierarchical relationships among the variables, where branch height indicates the degree of dissimilarity (distance) between clusters, with shorter branches representing greater similarity in correlation patterns.
