# Supplementary material for: Beyond HLA: an exploratory pilot study of non-HLA antibodies, HLA sensitization, and GSTT1 genotype in platelet transfusion refractoriness
Source: Front Immunol. 2026 Jul 30;17:1886771. doi: 10.3389/fimmu.2026.1886771 (PMC13469656; doi:10.3389/fimmu.2026.1886771)
Supplement: Supplementary file 2 [file Table1.docx]

**Supplementary Table S1. The corresponding target antigens and their full gene names of Non‑HLA antibodies**

| **Antigen** | **Gene Name** | **Exclusive to Immucor Luminex^©^ Assay** |
| --- | --- | --- |
| ACTIN | Actin | √ |
| AGRN | Agrin |  |
| APOL2 | Apolipoprotein L2 | √ |
| ARHGDIB | ARHGDIB |  |
| ATP5B | ATP Synthase F1 Subunit Beta | √ |
| CCP | Cyclic Citrullinated Peptide | √ |
| CD40 | CD40 molecule | √ |
| CGB5 | Chorionic Gonadotropin Subunit Beta 5 | √ |
| COLLAGEN I | Collagen I |  |
| COLLAGEN II | Collagen II |  |
| COLLAGEN III | Collagen III |  |
| COLLAGEN IV | Collagen IV |  |
| COLLAGEN V | Collagen V |  |
| COLLAGEN VI | Collagen VI | √ |
| CSF2 | Colony stimulating factor 2 | √ |
| CXCL11 | C-X-C Motif Chemokine Ligand 11 |  |
| CXCL9 | C-X-C Motif Chemokine Ligand 9 |  |
| DEXI | Dexamethasone-induced transcript | √ |
| EMCN | Endomucin | √ |
| ENO1 | Alpha-enolase |  |
| FAS | Fas Cell Surface Death Receptor | √ |
| FIBRONECTIN1 | Fibronectin 1 |  |
| FLRT2 | Fibronectin Leucine Rich Transmembrane Protein 2 |  |
| GAPDH | Glyceraldehyde-3-phosphate Dehydrogenase |  |
| GDNF | Glial Cell Derived Neurotrophic Factor |  |
| GSTT1 | Glutathione S-Transferase Theta-1 |  |
| HARS | Jo-1 | √ |
| HSPB1 | Heat Shock Protein Beta-1 | √ |
| Human Transferrin | Transferrin | √ |
| ICAM1 | Intracellular Adhesion Molecule 1 | √ |
| IFNG | Interferon Gamma |  |
| IL21 | Interleukin 21 | √ |
| IL8 | Interleukin 8, CXCL8 | √ |
| KRT18 | Cytokeratin 18 | √ |
| KRT8 | Cytokeratin 8 | √ |
| LGALS3 | Galectin 3 | √ |
| LGALS8 | Galectin 8 | √ |
| LMNA | Lamin-A/C |  |
| LPHN1 | Latrophilin 1 | √ |
| MYOSIN | Myosin |  |
| NCL | Nucleolin |  |
| P2RY11 | Purinergic Receptor P2Y11 | √ |
| PECR | Peroxisomal Trans-2-enoyl-CoA Reductase |  |
| PLA2R1 | Phospholipase A2 Receptor 1 |  |
| PRKCH | Protein kinase C, Eta |  |
| PRKCZ | Protein kinase C, Zeta |  |
| PTPRO | Receptor-type Tyrosine-protein Phosphatase U | √ |
| ROR1 | Receptor Tyrosine Kinase-Like Orphan Receptor 1 | √ |
| SHC3 | SHC Adaptor Protein 3 | √ |
| SNRPB2 | Small Nuclear Ribonucleoprotein Polypeptide B | √ |
| SNRPN | Small Nuclear Ribonucleoprotein Polypeptide N | √ |
| SSB | Sjogren Syndrome Antigen B | √ |
| STAT6 | Signal Transducer and Activator of Transcription 6 | √ |
| Thyroglobulin | Thyroglobulin | √ |
| TUBA1B | Tubulin Alpha 1b |  |
| TUBB | Tubulin Beta | √ |
| TUBULIN | Tubulin | √ |
| VCL | Vinculin | √ |
| VEGFA | Vascular Endothelial Growth Factor A | √ |
| VIM | Vimentin |  |
