# Supplementary material for: Beyond HLA: an exploratory pilot study of non-HLA antibodies, HLA sensitization, and GSTT1 genotype in platelet transfusion refractoriness
Source: Front Immunol. 2026 Jul 30;17:1886771. doi: 10.3389/fimmu.2026.1886771 (PMC13469656; doi:10.3389/fimmu.2026.1886771)
Supplement: Supplementary file 3 [file Table2.docx]

**Supplementary Table S2. Distribution of Non‑HLA Ab Positivity in PTR Patients and Healthy Blood Donors**

| **Characteristic** | **Controls** | **PTR** | ***p*-value***^a^* | **Adjusted-*p***  **(*p*_c_)** *^b^* |
| --- | --- | --- | --- | --- |
|  | (n=20) | (n=32) |  |  |
| ATP5B, n (%) |  |  | 1.000 | 1.000 |
| Negative | 19 (95%) | 31 (97%) |  |  |
| Positive | 1 (5.0%) | 1 (3.1%) |  |  |
| CSF2, n (%) |  |  | <0.001 | 0.009 |
| Negative | 20 (100%) | 18 (56%) |  |  |
| Positive | 0 (0%) | 14 (44%) |  |  |
| ENO1, n (%) |  |  | 0.283 | 0.781 |
| Negative | 18 (90%) | 24 (75%) |  |  |
| Positive | 2 (10%) | 8 (25%) |  |  |
| GDNF, n (%) |  |  | 0.276 | 0.781 |
| Negative | 20 (100%) | 29 (91%) |  |  |
| Positive | 0 (0%) | 3 (9.4%) |  |  |
| GSTT1, n (%) |  |  | 0.009 | 0.075 |
| Negative | 20 (100%) | 23 (72%) |  |  |
| Positive | 0 (0%) | 9 (28%) |  |  |
| ICAM1, n (%) |  |  | 1.000 | 1.000 |
| Negative | 19 (95%) | 31 (97%) |  |  |
| Positive | 1 (5.0%) | 1 (3.1%) |  |  |
| IFNG, n (%) |  |  | 0.517 | 0.781 |
| Negative | 20 (100%) | 30 (94%) |  |  |
| Positive | 0 (0%) | 2 (6.3%) |  |  |
| IL21, n (%) |  |  | 0.517 | 0.781 |
| Negative | 20 (100%) | 30 (94%) |  |  |
| Positive | 0 (0%) | 2 (6.3%) |  |  |
| IL8, n (%) |  |  | 0.276 | 0.781 |
| Negative | 20 (100%) | 29 (91%) |  |  |
| Positive | 0 (0%) | 3 (9.4%) |  |  |
| LGALS3, n (%) |  |  | 0.517 | 0.781 |
| Negative | 20 (100%) | 30 (94%) |  |  |
| Positive | 0 (0%) | 2 (6.3%) |  |  |
| PECR, n (%) |  |  | 0.551 | 0.781 |
| Negative | 18 (90%) | 31 (97%) |  |  |
| Positive | 2 (10%) | 1 (3.1%) |  |  |
| PRKCZ, n (%) |  |  | 0.695 | 0.909 |
| Negative | 16 (80%) | 28 (88%) |  |  |
| Positive | 4 (20%) | 4 (13%) |  |  |
| SSB, n (%) |  |  | 1.000 | 1.000 |
| Negative | 19 (95%) | 30 (94%) |  |  |
| Positive | 1 (5.0%) | 2 (6.3%) |  |  |
| STAT6, n (%) |  |  | 0.276 | 0.781 |
| Negative | 20 (100%) | 29 (91%) |  |  |
| Positive | 0 (0%) | 3 (9.4%) |  |  |
| Thyroglobulin, n (%) |  |  | 1.000 | 1.000 |
| Negative | 17 (85%) | 26 (81%) |  |  |
| Positive | 3 (15%) | 6 (19%) |  |  |
| TUBULIN, n (%) |  |  | 0.517 | 0.781 |
| Negative | 20 (100%) | 30 (94%) |  |  |
| Positive | 0 (0%) | 2 (6.3%) |  |  |
| VEGFA, n (%) |  |  | 0.517 | 0.781 |
| Negative | 20 (100%) | 30 (94%) |  |  |
| Positive | 0 (0%) | 2 (6.3%) |  |  |

^a^ Fisher’s exact test; ^b^ Using Benjamini-Hochberg (BH) method to adjust *p*-values (*p*_c_). PTR, platelet transfusion refractoriness; Non-HLA Ab, non-HLA antibody.
