# Supplementary material for: Beyond HLA: an exploratory pilot study of non-HLA antibodies, HLA sensitization, and GSTT1 genotype in platelet transfusion refractoriness
Source: Front Immunol. 2026 Jul 30;17:1886771. doi: 10.3389/fimmu.2026.1886771 (PMC13469656; doi:10.3389/fimmu.2026.1886771)
Supplement: Supplementary file 4 [file Table3.docx]

**Supplementary Table S3. Prevalence of *GSTT1* homozygous deletion in non-iPTR, iPTR Patients and Healthy Blood Donors**

| **Characteristic** | **Controls** | **Non-iPTR** | **iPTR** | **PTR** | *p*-value*^a^* |
| --- | --- | --- | --- | --- | --- |
|  | (n = 20) | (n= 16) | (n= 16) | (n= 32) |  |
| *GSTT1* homozygous deletion, n (%) | 10 (50%) | 9 (56%) | 7 (44%) | 16 (50%) | 0.919 |
| gender, n (%) |  |  |  |  | 0.965 |
| female | 14 (70%) | 10 (63%) | 11 (69%) | 21 (66%) |  |
| male | 6 (30%) | 6 (38%) | 5 (31%) | 11 (34%) |  |
| age, Median (Q1, Q3) | 43 (24, 51) | 59 (49, 64) | 57 (53, 66) | 57 (53, 65) | <0.001 |
| Pregnancy history, n (%) | 9 (45%) | 9 (56%) | 11 (69%) | 20 (63%) | 0.487 |

^a^ Pearson’s Chi-squared test; Kruskal-Wallis rank sum test; Fisher’s exact test; iPTR, immune platelet transfusion refractoriness; PTR, platelet transfusion refractoriness; *GSTT1*, Glutathione S-Transferase theta-1.
